# The effect of exercise modality on age-related changes observed during running

**DOI:** 10.1186/s11556-022-00302-3

**Published:** 2022-09-17

**Authors:** Brianne Borgia, Janet S. Dufek, Kara N. Radzak, Julia Freedman Silvernail

**Affiliations:** Department of Kinesiology and Nutrition Sciences, University of Nevada, Las Vegas, Las Vegas, NV USA

**Keywords:** Gait, Kinematics, Kinetics, Aging, Physical activity

## Abstract

**Introduction:**

With the increase in participation by older adults in endurance events, research is needed to evaluate how exercising throughout the lifespan can affect the aging process regarding gait and mobility. The purpose of this study was to determine how the type of exercise modality one participates in will affect age-related declines observed during running.

**Methods:**

Fifty-six individuals between the ages of 18–65 who considered running, resistance training or cycling/swimming as their primary form of activity participated in this study. Kinematics were captured using a 10-camera motion capture system while participants ran at a controlled pace of 3.5 m/s (± 5%) over a 10-m runway with force platforms collecting kinetic data. Eight successful trials were chosen for analysis. A one-way ANOVA assessed differences in mean kinematic and kinetic variables of interest between physical activity groups (α = 0.05).

**Results:**

Older resistance trainers exhibited greater maximal knee power compared to older runners. No other group differences were observed.

**Conclusion:**

Despite type of exercise modality, regularly participating in exercise has positive effects. This is evident through the preservation of the function of the lower extremity with age, specifically function of the ankle, and its contribution to healthy movement patterns.

## Introduction

The importance of an active lifestyle has been well defined for general health [1] as one ages, but less is known about the influence on movement health. Neuromuscular changes occur with aging, contributing to a decline in mobility and performance [2–4]. A central theory to the underlying cause of gait and mobility limitations with aging is a decrease in muscle function, specifically age-related muscle loss [2, 5, 6]. Although encouraging evidence suggests that physical activity can attenuate and possibly reverse aging related muscle loss [7–9]. However, additional factors like strength, balance, joint mobility, and fatigability can together lead to dynamic gait adaptations. As the participation by older adults in endurance events continues to increase [10, 11], research is needed to evaluate how exercising later into life can affect the aging process and the musculoskeletal health of these individuals.

Sedentary aging adults see a reduction in joint motion between 10–40%, depending on the body part, and a reduction in muscle mass by 40% [12], resulting in decreases in mobility and altered gait mechanics. These alterations are evident through decreases in stride length, joint angular displacement [13, 14], and joint torque and power [15–17] that have been observed in older adults compared to younger individuals during walking. Changes in movement may be part of a compensation strategy for age-related changes categorized by a distal-to-proximal shift where older adults increase the use of proximal joints compared to distal joints during gait [15, 18]. Several studies have reported supportive findings including that older adults exhibit reduced ankle range of motion and plantar flexor power [15, 17, 19, 20], and increased hip range of motion [21, 22] and power generated at the hip [15, 19, 20] during gait compared to young adults. This compensation strategy has been observed during both walking and running [21–25]. These altered gait mechanics observed with aging may also lead to changes in stability and balance, thereby increasing the already elevated risk of injury [26, 27].

Resistance and aerobic exercises increase muscle strength, aerobic capacity, and bone density [28–31] all of which to transfer to functional tasks such as gait. Research comparing active older individuals to their sedentary peers found that many of the previously mentioned declines associated with aging are the result of a sedentary lifestyle [32–34] or disuse [34]. Intervention studies have shown that beginning participation in exercise training programs can minimize age related changes and contribute to improvements in health [1]. However, in most aging studies comparing individuals who are already regularly physically active, the exercise profiles (e.g., preferred exercise modality) of the participants are often vague or not reported.

Different exercise modalities provide different benefits in terms of muscle strength [35, 36], balance [37, 38], endurance [39], and activities of daily living [40]. Therefore, the purpose of this study was to determine how the type of exercise modality one participates in will affect age-related declines observed during running. We focused on running endurance exercise, non-running (swim/cycling) endurance exercise, and resistance training (RT) for the scope of this project. Our general hypothesis was that exercise modality would influence age-related declines, as defined by the contribution of lower extremity joints (joint angular motion, joint moments, and joint power) to gait performance. Previous studies have observed a distal-to-proximal shift in contribution from the lower extremity during stance in older adults compared to younger adults [22, 23, 41–43]. Accordingly, we hypothesized that older active individuals, regardless of exercise mode, would have lower ankle contributions than younger runners. As different types of exercise provide different functional benefits, we expected older individuals participating in different exercise modalities to exhibit different gait patterns. Due to the fact that running endurance training can help slow age-related gait declines [8], it was hypothesized that older runners would exhibit less of a distal to proximal shift (i.e. greater contribution from the ankle) than both resistance trainers and swim/cyclists.

## Methods

### Participants

**Fig. 1:**
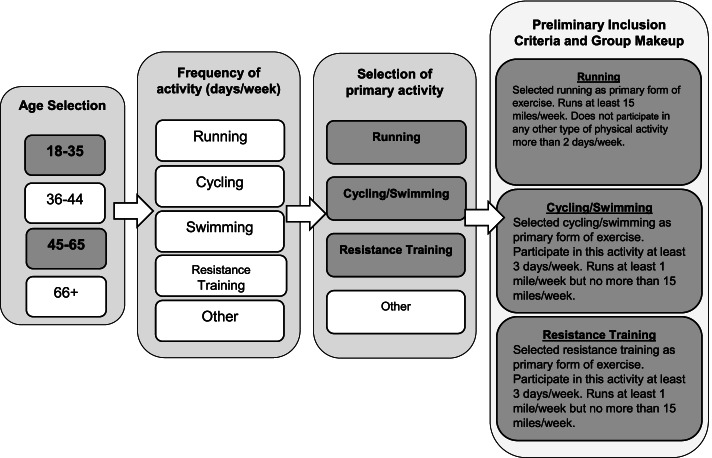
Basic flow of pre-screening survey and description of preliminary inclusion criteria and group makeup

Fifty-six individuals between the ages of 18–65 who regularly participated in one of three different exercise modalities as their primary form of physical activity were recruited for this study, creating four groups of 14 participants. Data from the literature were used to estimate sample size for a minimum statistical power of 80% with an alpha level of 0.05. Dependent variables utilized in the power analysis included sagittal plane hip, knee, ankle joint kinetics [41, 43, 44]. The projected sample size to obtain a moderate effect size was approximately 10–14 participants per group for this between group comparisons. A pre-screening survey was created to help determine initial eligibility. The basic flow of the survey and preliminary inclusion criteria can be found in Fig. 1. Prospective participants were asked questions about their daily physical activity including the type and frequency of activity, their primary form of exercise, and running history. Participants who fell into one of the three activity groups were asked to participate in the study. Additional inclusion criteria required being free of lower extremity injury for the past 6 months and at the time of testing, as well as having no history of lower extremity surgery that may affect their gait. Based on their completion of physical activity readiness and health history questionnaires, all participants were considered low risk for participating in physical activity according to the American College of Sports Medicine. The protocols for this study were approved by University Institutional Review Board (1,346,396–4) and all participants provided written informed consent prior to participation.

To proceed with data collection, participants were asked to complete questionnaires verifying their answers from the prescreening survey. This included type(s) of activity they participated in, frequency of activity, and selection of their primary form of physical activity. Further information was provided regarding the participants’ running training. As shown in Table 1, the information collected included weekly running mileage, self-reported training pace, runs per week, years of running, and days per week participating in their primary form of physical activity. Four distinct groups were created: Older Runners, Younger Runners, Resistance Training, and Swim/Cycling. Participants were placed into their respective age running group if they reported running at least 15 miles/week, participated in no other type of physical activity more than 2 days/week, and selected running as their primary form of activity. The resistance training group included participants who participated in resistance training at least three days/week, ran at least 1 mile per week, but not more than 15 miles, and selected resistance training as their primary activity. Those who met the criteria for the swim/cycling group reported engaging in these activities at least three days/week, reported running at least one mile/week but no more than 15 miles, and using either of these activities as their primary form of exercise.

**Table 1:** Participant demographics

	Running		Resistance Training	Cycle/Swim
	Young	Older		
Sex	9F, 5 M	7F, 7 M	8F, 6 M	7F, 7 M
Age(yrs)	26.5 (6.68)	53.82 (5.73)^*^	50.00 (3.88)^*^	51.67 (6.71)^*^
Mass (kg)	62.21 (9.94)	68.06 (12.80)	67.19 (9.48)	72.35 (13.22)
Height (m)	1.72 (0.13)	1.70 (0.16)	1.70 (0.09)	1.71 (0.11)
BMI (kg/m2)	20.99 (2.53)	23.22 (1.63)	23.19 (1.90)	24.55 (3.15)
Body Fat (%)	17.18 (8.93)	23.28 (5.72)	20.44 (4.86)	23.69 (10.22)
Miles/week	30.33 (13.19)	31.67 (12.49)	7.18 (5.23)†	12.25 (5.19) †
Self-reported pace (min mile-1)	8.02 (1.18)†	9.52 (1.62)^*^	9.48 (1.41)	10.33 (1.35)^*^
Days/week	5.00 (1.21)	4.75 (1.06)	3.92 (1.08)	4.67 (1.50)
Runs/week	5.00 (1.21)	4.75 (1.06)	2.00 (0.74)†	2.42 (0.67)†
Running experience (yrs)	7.58 (4.17)	19.33 (12.99)	19.40 (12.49)	20.45 (13.29)

Mean (standard deviation); *m* meters, *kg* kilogram, *BMI* body mass index, days/week: number of days participating in respective primary activity

^*^ significantly different from young runners

^†^ significantly different from older runners; (*p* ≤ 0.05)

### Experimental set up and protocol

The lab space consists of a 10-m runway with three embedded force platforms (AMTI, Watertown, MA) surrounded by a ten-camera three-dimensional motion capture system (Vicon Inc., Oxford, UK). Two photoelectric timing gates placed 4 m apart on either side of the force platforms quantified running velocity.

Participants were provided with neutral laboratory shoes and instructed to wear tight fit clothing. Anthropometric data, including height, weight, and body fat percentage (inBody 770, Cerritos, CA), were recorded. Retroreflective markers were placed on the pelvis and bilaterally on the thigh, shank and foot [45]. Prior to data collection, participants were allowed to perform a 5-min warm up at a self-selected pace, whether that be on a treadmill or paces around the laboratory. Following completion of the warmup, participants were instructed to perform running trials at a controlled pace of 3.5 m.s-1 ± 5% while kinematic and kinetic data were recorded at 200 and 1000 Hz, respectively. A controlled pace of 3.5 m.s-1 ± 5% was selected to allow interstudy comparisons as this is a common range used in studies evaluating running biomechanics of older runners [22, 46–48]. Eight successful trials were collected. A successful trial is one during which the right foot landed completely on the force platform with no signs of targeting or alterations in gait. To prevent targeting, participants were not informed of the location of the force platforms and their starting position was adjusted by a research team member to ensure a natural stride.

### Data analysis

Marker trajectories and ground reaction forces were exported to Visual 3D (C-Motion, Inc., Rockville MD) where they were filtered using a 4th order, zero lag, low-pass Butterworth filter with a cut off frequency of 12 Hz and 50 Hz, respectively. Stance phase was defined using filtered ground reaction forces based on when forces rose above and fell below a 20 N threshold. Static trials were used to define anatomical coordinate systems for the rearfoot, shank, and thigh with coordinate systems defined based on recommendations of the International Society of Biomechanics Joint [49]. Joint angles were calculated at the knee and ankle as rotations of the distal segment relative to the proximal segment using an XYZ Cardan rotation sequence corresponding to flexion/extension, ab/adduction, and axial rotation. Joint moments were calculated using a standard inverse dynamics approach. Sagittal plane joint angles, moments, and powers were calculated at the ankle, knee, and hip during the stance phase of gait and exported to a custom Matlab (Mathworks, Natick, MA) program where additional variables of interested were calculated and averaged for all trials for each participant. These included angles at initial contact, peak joint angles, moments, and power, and peak ground reaction forces.

### Statistical analysis

A one-way ANOVA assessed differences in mean kinematic and kinetic variables between all activity groups. An alpha level of 0.05 was used to indicate statistical significance. In the event of a significant omnibus F-test, post-hoc pairwise comparisons were conducted using an LSD correction to determine where differences occurred. Cohen’s d effect size calculations were also used to assess group differences in lower extremity mechanics. All statistical tests were performed using Statistical Package for the Social Sciences (SPSS, IMB Corp, Armonk, NY), version 25.

## Results

Descriptive statistics for group demographics can be found in Table 1. Exercise groups consisting of older individuals were similar in age, mass, height, body mass index, and percent body fat. Additionally, all individuals participated in their primary form of exercise a similar number of days per week and had comparable years of running experience. The fourteen young runners who participated in this study were matched to older runners for weekly mileage and were similar in all other demographic characteristics aside from age and self-reported training pace. Self-reported training pace was significantly different between exercise modality groups, *F*(3,52) = 5.22, *p* = 0.004, in that the training pace of younger runners was faster than both older runners (*p* = 0.047) and the swim/cycling group (*p* = 0.02).

Mean kinematic and kinetic variables of interest can be found in Table 2. These variables included hip, knee, and ankle angles during the stance phase of gait. Kinetic variables included hip, knee and ankle joint moments, power, and work. Maximum knee power was significantly different between exercise modality groups, *F*(3,52) = 3.394, *p* = 0.025. Post hoc analysis revealed that older runners generated less knee power compared to the resistance training group during the stance phase of gait *(p* = 0.17, d = 0.98). No other group differences were observed.

**Table 2:** Mean (standard deviation) of lower extremity kinematics and kinetics during the stance phase of gait

	Running		Resistance Training	Swim/Cycle
	Young	Older		
*Kinematics (°)*				
Ankle IC	1.71 (5.10)	3.52 (4.42)	3.60 (6.26)	2.48 (3.64)
Ankle Peak	21.17 (1.65)	21.94 (1.68)	22.36 (3.50)	21.06 (2.03)
Ankle ROM	19.46 (4.93)	18.42 (3.81)	22.69 (11.75)	18.58 (3.94)
Knee IC	-17.39 (2.38)	-18.80 (3.10)	-17.44 (2.91)	-17.29 (4.74)
Knee Peak	-41.69 (2.38)	-41.63 (2.63)	-42.89 (4.89)	-40.68 (3.88)
Knee ROM	24.30 (2.84)	22.83 (2.79)	26.70 (7.69)	23.39 (3.95)
Hip IC	46.10 (4.77)	46.83 (6.60)	45.94 (6.60)	45.66 (5.69)
Hip ROM	44.74 (5.80)	45.27 (4.38)	45.87 (6.15)	45.25 (8.47)
*Kinetics*				
Peak vGRF (N/BW)	2.52 (0.16)●	2.34 (0.20)	2.60 (0.37)●	2.44 (0.26)
Peak PF moment (Nm/kg)	-2.74 (0.30)	-2.54 (0.33)	-2.76 (0.58)	-2.71 (0.55)
Peak KE moment (Nm/kg)	2.41 (0.37)●	2.15 (0.21)	2.71 (0.91)	2.43 (0.51)
Peak HE moment (Nm/kg)	-2.50 (0.45)	-2.66 (0.78)	-2.84 (1.35)	-2.77 (0.98)
Max ankle power (W/kg)	14.26 (2.79)	12.32 (2.20)	14.61 (3.39)	14.00 (3.15)
Max knee power (W/kg)	5.34 (1.10)	4.80 (0.59)	6.83 (2.87) *	5.75 (1.56)●
Max hip power (W/kg)	3.76 (1.25)	4.65 (1.36)	4.55 (2.27)	4.19 (1.26)
Positive ankle work (J/kg)	0.14 (0.03)	0.14 (0.2)	0.15 (0.04)	0.15 (0.03)
Positive knee work (J/kg)	0.05 (0.01)	0.05 (0.01)	0.06 (0.02)	0.05 (0.02)
Positive hip work (J/kg)	0.04 (0.02)	0.06 (0.02)	0.05 (0.01)	0.05 (0.01)

*IC* initial contact, *ROM* range of motion, *vGRF* vertical ground reaction force, *PF* plantarflexor, *KE* knee extension, *HE* hip extension, *N* newtons, *BW* body weight, *Nm* newton meters, *kg* kilograms, *W* watts, *J* joules

^*^ significant difference between respective group and older runners, (*p* ≤ 0.05)

● denotes large effect size (≥ .8) between respective group and older runners

## Discussion

The purpose of this study was to investigate how the type of exercise modality one participates in is related to the age-related declines observed during running. Contrary to our hypotheses, we did not observe differences between older runners and young runners, nor between older active individuals, the RT group or the Swim/Cycle group, and young runners, respectively. Interestingly, however, we did observe a difference between older active adults in that resistance trainers generated more knee power compared to older runners during the stance phase of gait.

The findings from this study suggest that while the type of exercise modality may not matter, remaining physically active later into life preserves movement patterns similar to younger individuals. However, our results do indicate that the type of exercise may be influential to some extent, as we did find differences between the older adult groups. Older adults in our resistance training group generated greater knee power compared to older runners. A likely strategy for older adults is the utilization of more proximal joints during gait, however while there were differences at the knee joint, maximum ankle power was similar. Previous studies have found performance differences between those participating in strength modalities and aerobic modalities, reporting a greater decrease in performance in those participating in aerobic exercise [50–52]. While maximal strength was not assessed using a designated device (i.e. isokinetic device), a possible explanation for the greater knee power observed in our resistance training group is a greater preservation of muscle properties from participating in strength activity as their primary form of exercise [53]. According to a recent study on master athletes [54], lower extremity muscles exhibit an age-related slowing of contraction onset. Age-related increases in contractile times were observed in endurance athletes as well as non-athletes; however, power athletes maintained shorter contraction times with age [54], demonstrating the important of high-intensity exercise to the slowing of age-related skeletal muscle decline.

An alternative explanation for the increased load on the knee joint in our RT group may be the result of greater peak vGRF, as suggested by a large effect size (*d* = 0.9). The greater vGRF may also be the result of shorter stride length as these two variables are highly correlated 41. Previously, older runners have exhibited shorter stride length compared to young runners at both self-selected and controlled velocities41, however we did not include spatiotemporal variables in our analysis. While not statistically significant, older adults in the swim/cycle group also generated greater knee power compared to the older runners (*d* = 0.8), however these two groups had similar peak vGRFs. Previous studies have shown greater knee power in forefoot strike runners compared rearfoot strike runners [55, 56] however we did not control or identify strike pattern in our study. Lastly, we recognize that experience of the running group may allow those individuals to move more efficiently and therefore have a more optimal distribution of joint power, leading to lower knee power compared to RT. However, few studies assessing the influence of running exposure have found that it does not appear to influence running mechanics in distance runners [57] or runners over the age of 50 [58].

Although previous literature investigating the mechanics of older and younger runners report differences in both kinematics [22, 23, 41–43] and kinetics [8, 21–24, 41, 43, 46, 59], we observed no differences between our groups of runners. A common observation in older runners is alterations in joint range of motion throughout the gait cycle. When running at a controlled pace, older runners exhibit range of motion modifications at the ankle, knee, and hip [22, 23, 41–43] that may be in part due to the age-related decreases in musculoskeletal strength and flexibility. However, we did not observe any of these kinematic changes among the older runners in our study, nor did we observe any differences in joint kinetics between our running groups. Because our groups ran similarly, it is possible that exercise, regardless of modality, is a protective mechanism to age-related gait declines. Devita and Hortobagyi [15] reported that during walking, older adults exhibit a distal to proximal shift in joint powers during walking. While a similar compensation strategy has been reported in older adults during running [21, 23, 24], there are inconsistent findings within the current literature. Kulmala et al. [23] reported increased power generation from the hip extensors in older runners compared to young runners, as well as decreases in peak plantarflexion moments and ankle power generation. Alternatively, Fukuchi et al. [43] observed no differences in joint kinetics between older and young runners. While the observed similarities between our running groups did not support our hypothesis, this is not entirely surprising when we look at the characteristics of our runners. The participants making up our older and younger runners were matched for weekly mileage running ~ 30 miles/week. Additionally, these groups ran a similar number of days per week. Previous literature comparing older and younger runners who were matched for weekly mileage, training load, or ran a minimum of 10 miles/week reported fewer differences and more group similarities between age groups [8, 22, 42].

One of the driving forces behind this study was to recruit participants that represented active older adults and to confidently report and quantify their participation in exercise given our resources. In doing so, our groups of participants are homogenous in nature which likely influenced our results. While we realize this can be viewed as a limitation, we believe that the narrow inclusion criteria were important to answer our research question. For this study we recruited individuals between the ages of 45–65, with the oldest participants included being 61 years old and the average age of all our older participants being 51 years old. We acknowledge that this is younger than similar studies including older adults making comparisons difficult and that it can be seen as a limitation. However, the primary focus of this study was not to compare older versus younger individuals, rather to investigate how specific types of physical activity may contribute to the prevention or postponement of gait declines often experienced with age. Given that age-related changes that may affect gait can begin as early as in your 20s [60], the older individuals included in this study represent a population who may have already begun experiencing age-related gait adaptations. Even though our older participants were on average in their sixth decade of life, it may be that they were too young to observe the changes previously reported in the literature, and instead, are representative of a middle-aged population.

## Conclusion

In our study, regular exercise had positive effects on preserving lower extremity joint and muscle function with age, specifically ankle function, and contributing to healthy movement patterns, regardless of exercise. Our findings highlight the need to better describe participants regarding the type and amount of physical activity they participate in when conducting research on active populations. The amount of regular physical activity by our participants was enough to mitigate the age-related distal to proximal shift, however it is possible our participants were not old enough. While this shift has been observed in active older populations, the onset of this age-related decline, and its relationship to physical activity, requires further investigations.

## Data Availability

NA.
